# ORASPORT study protocol: a prospective observational study on the impact of endurance physical activity on oral microbiology and immunology

**DOI:** 10.3389/froh.2025.1692359

**Published:** 2025-12-05

**Authors:** Morgane Ortis, Margaux Dubois, Valérie Bougault, Kévin Legueult, Julien Fassy, Bernard Mari, Marie-France Bertrand, Laurence Lupi, Alain Doglio

**Affiliations:** 1 Laboratoire MICORALIS, Faculté de Chirurgie Dentaire, Université Côte d’Azur, Nice, France; 2 Institut de Médecine bucco-dentaire, Centre Hospitalier Universitaire de Nice, Pôle Odontologie, Nice, France; 3 Laboratoire LAMHESS, Campus Science du sport, Université Côte d’Azur, Nice, France; 4 Département de Santé Publique, Université Côte d'Azur, Centre Hospitalier Universitaire de l’Archet, Nice, France; 5 Institut de Pharmacologie Moléculaire et Cellulaire (IPMC), Centre National de la Recherche Scientifique, CNRS, UMR 7275, Valbonne, France; 6 Unité de Thérapie Cellulaire et Génique (UTCG), Centre Hospitalier Universitaire de Nice, Nice, France

**Keywords:** oral health, oral microbiota, cytokines, endurance sports, protocol

## Abstract

The oral cavity offers a non-invasive window into systemic health. Elite athletes often present with poor oral health, potentially linked to high physiological stress, psychological strain, and specific dietary habits. This study explores the relationship between physical activity and oral health, with a particular focus on identifying key changes in the oral microbiota and salivary immune profiles. The main objective is to compare the composition of oral microbiota and the levels of salivary cytokines among young healthy individuals with varying levels of endurance physical activity. The ORASPORT study is a prospective cross-sectional non-interventional study involving 200 participants recruited in a single center. The participants are healthy young individuals aged 18–30, with or without regular endurance physical activity. They will be divided into three groups according to their physical activity level, following international guidelines set by the World Health Organization (WHO). For each consenting participant, a standardized oral health assessment will be conducted by a dentist to establish a comprehensive oral health profile. Participants will also complete a detailed questionnaire to document individual data on physical activity, dietary habits, and overall health status. Additionally, oral biological samples, including saliva and gingival smears, will be collected for microbiological analysis and cytokine detection. Microbiological analysis of all oral samples will be performed using high-throughput qPCR with Biomark HD™ technology, to simultaneously detect 40 oral bacterial species, selected to represent both commensal and pathogenic oral flora, as well as the presence of 8 Human Herpesviruses (HHVs). Concurrently, the salivary concentrations of 9 inflammatory cytokines will be quantified using Meso Scale Discovery® multiplex immunoassays. Statistical analysis will include both univariate and multivariate models to identify correlations between physical activity, oral health parameters, and microbial-immune profiles. This study protocol integrates clinical, microbiological, and immunological analysis to identify non-invasive biomarkers of oral health and physical performance. This study complies with French bioethics regulations (Jardé Law) and with data protection under European GDPR requirements. It received French ethical approval from the Committee for the Protection of Persons in February 2023. Findings will be shared through publications in peer-reviewed journals and presentations at international conferences.

**Clinical trial number:**

NCT05765422.

## 1 Introduction

The oral cavity reflects systemic health, serving as a mirror of both wellness and disease. As an easily accessible and non-invasive site, it offers unique opportunities for investigating systemic conditions (1). A bidirectional relationship exists between oral health and overall health: oral manifestations can indicate underlying systemic diseases (e.g., diabetes, cancer, infections), while poor oral health can, in turn, exacerbate systemic conditions such as diabetes, obesity, and cardiovascular diseases (2–5). Physical activity is essential for maintaining good health throughout life. However, recent data highlight a significant public health concern: in France, only 5% of adults achieve the recommended levels of physical activity, and more than one-third report a combination of sedentary behavior and low physical activity [from the French Agency for Food, Environmental and Occupational Health & Safety (ANSES)] (6). Sedentary behavior is a major risk factor for numerous non-communicable diseases, including cardiovascular diseases, diabetes, and certain cancers (7). Moreover, recent studies have also linked insufficient physical activity to an increased risk of periodontal disease, underscoring the systemic impact of lifestyle factors on oral health (8). However, despite their high levels of physical activity, elite athletes often experience compromised oral health, characterized by a high prevalence of dental caries, periodontitis, trauma, and xerostomia (9, 10). Although research on this issue remains limited, several contributing factors have been suggested, including the frequent consumption of acidic energy drinks, gastroesophageal reflux, bruxism due to psychological stress, dehydration, and inadequate oral hygiene. Additionally, the use of mouthguards in contact sports has been shown to affect the oral microbiome, thus causally leading to microbial dysbiosis (11). A bidirectional relationship between oral health and physical performance has also been observed. Periodontitis, for example, has been associated with lower physical performance scores, suggesting that periodontal disease may serve as an indicator of impaired physical fitness (12). Interestingly, intense physical exertion and stress can depress salivary immune function, making elite athletes more susceptible to minor infections, particularly following severe exercise (13). Several studies have described associations between high-intensity sports and immune dysregulation (14, 15). At the oral level, these alterations manifest as changes in salivary protein composition and the expression of antimicrobial chemokines (15, 16).

Oral health is closely associated with the composition and balance of the oral microbiota. This complex microbial ecosystem has emerged as a promising field of research, providing valuable insight into various health states (17). The oral cavity is a reservoir of a dense and diverse microbiota composed of thousands of bacterial, viral, and fungal species (18, 19). Under healthy conditions and with good oral hygiene, this microbial community maintains a stable composition that supports oral and systemic health. However, numerous factors that encompass oral hygiene practices, diet, psychological stress, systemic diseases, and smoking may disturb this fragile microbial balance. Similarly, to the gut microbiota dysbiosis, which has been associated with intestinal disorders (20, 21), autism (22), viral infections (23), and dietary changes (24, 25), alterations in the oral microbiome have been implicated in the onset of local conditions such as dental caries, candidosis, and periodontal diseases (26). Moreover, a growing body of evidence links oral dysbiosis to systemic diseases, including diabetes (27), Alzheimer's disease (28), Rheumatoid Arthritis (29, 30). In addition, the role of oral microbiota in carcinogenesis has gained attention, with studies highlighting its potential involvement in various cancers, including oral cancer (31, 32).

Previous studies have demonstrated that significant microbiological differences can be detected even with small sample sizes (33). Most investigates in this field involve approximately 20 participants, and the study conducted by Lamb et al. demonstrates statistically significant differences with as few as 10 participants per group (34). While the impact of physical exercise on the gut microbiota has been increasingly studied, its effects on the oral microbiota remain poorly understood (35). This study aims to explore the relationship between oral microbiota, immune profiles, and endurance training by examining immune markers and microbial signatures from oral samples. The primary objective of this cross-sectional study is to assess the oral microbiota and inflammatory status of healthy individuals engaged in varying levels of physical activity. Ultimately, this research seeks to identify specific microbiological and immunological signatures associated with both sedentary behavior and physical activity.

## 2 Methods and analysis

### 2.1 Outcomes

This cross-sectional study explores the relationship between oral microbiota composition, immune function, and physical activity levels in young, healthy individuals. The primary objective is to identify microbial and immunological oral signatures associated with varying levels of endurance activity. The microbial targets include a panel of commensal and pathogenic bacterial species, as well as human herpesviruses. By profiling the oral microbiota and salivary immune markers across a spectrum of physical activity, from sedentary to highly active individuals, we seek to identify potential microbial and immunological biomarkers associated with physical activity levels. All targeted microorganisms, cytokines, and recorded parameters are summarized in Table 1.

**Table 1:** Study outcomes.

Principal study endpoints			
Commensal Bacteria	Pathogenic Bacteria	Human Herpes Viruses	Inflammatory Cytokines
*Streptococcus oralis*	*Porphyromonas gingivalis,*	HSV-1,	IFN-*γ*,
*Streptococcus gordonii*	*Streptococcus mutans,*	HSV-2,	IL-1β,
*Streptococcus salivarius,*	*Aggregatibacter*	EBV,	IL-2,
*Streptococcus sanguinis,*	*actinomycetemcomitans,*	CMV,	IL-4,
*Gemella haemolysans,*	*Corynebacterium matruchotii,*	VZV,	IL-6,
*Capnocytophaga gingivalis,*	*Fusobacterium nucleatum,*	HHV-6a,	IL-10,
*Micrococcus luteus,*	*Tannerella forsythia,*	HHV-6b,	IL-12p70,
*Rothia mucilaginosa,*	*Dialister pneumosintes,*	HHV-7,	IL-17A,
*Actinomyces naeslundii,*	*Filifactor alocis,*	HHV-8	TNF-α
*Granulicatella adiacens,*	*Treponema denticola,*		
*Veillonella parvula,*	*Prevotella intermedia,*		
*Eikenella corrodens,*	*Prevotella denticola,*		
*Actinomyces massiliensis*	*Prevotella melaninogenica,*		
	*Prevotella nigrescens,*		
	*Centipeda periodontii,*		
	*Campylobacter rectus,*		
	*Klebsiella pneumoniae,*		
	*Selenomonas sputigena,*		
	*Pseudomonas aeruginosa,*		
	*Haemophilus parainfluenzae,*		
	*Peptostreptococcus anaerobius,*		
	*Eubacterium nodatum,*		
	*Propionibacterium propionicum,*		
	*Parvimonas micra,*		
	*Megasphaera micronuciformis,*		
	*Porphyromonas endodontalis*		
Secondary study endpoints			
Caries	Gingival status	Tooth wear	Extra
• DMF index (WHO criteria)	• Loe and Silness Index (LSI)	• Absence	• Orthodontic retainer
• ICDAS system	• Plaque Index (PI)	• Presence	• MIH
	• Gingival recession (Miller)		• Fluorosis
			• Trauma
			• Tartar
Other study parameters			
• Socio-demographic factors			
• Salivary flow			
• Sport habits			
• Diet			
• General health status			
• Oral health status			
• Oral Health Impact Profile-14 (OHIP-14)			

The study's secondary objective is to assess and compare the dental health of participants across the different activity groups. Clinical oral health assessments will include the evaluation of dental caries, tooth wear, periodontal status, and other relevant clinical indicators. To reduce potential confounding biases, data will also be collected through structured questionnaires on diet, supplement use, general and oral quality of life (OHIP-14), socio-demographic characteristics, medication intake, lifestyle habits, healthcare (medical and dental), and oral hygiene practices.

### 2.2 Study design and study population characteristics

ORASPORT is a single-center, cross-sectional, prospective pilot study. A total of 200 participants aged 18 to 30 years will be enrolled over a 30-month period.

Participants will be primarily recruited from the Sports and Dental Departments of Côte d'Azur University (France), as well as through local sports competitions. Additional recruitments will take place through researcher networks, university posters, and social media platforms. The inclusion criteria are: (i) age between 18 and 30 years old, (ii) male or female, and (iii) if practicing a physical activity, the primary sport must be an endurance activity (i.e., cycling or running).

Participants (*n* = 200) will be classified into three groups according to their physical activity levels: (i) Group 1 (*n* = 50): participants who do not meet the WHO recommendations, (ii) Group 2 (*n* = 100): participants who meet the WHO recommendations, defined as engaging in 150 to 300 min per week of moderate-intensity physical activity, or 75 to 150 min per week of vigorous-intensity physical activity, and (iii) Group 3 (*n* = 50): participants who exceeded the WHO recommendations. The larger size of Group 2 was chosen to account for the expected higher heterogeneity among participants meeting the WHO recommendations compared with those not meeting or exceeding them. The sex of the participants will be balanced as much as possible across the three groups during recruitment to account for potential differences in oral microbiota composition and immune profiles between men and women.

The sample size was not determined through a formal power calculation, as this is an exploratory pilot study designed to generate preliminary data and refine the methodology for a future large-scale investigation. Instead, the proposed sample size was estimated based on feasibility considerations. This number was deemed sufficient to capture variability within and between groups while remaining achievable within the study period. The results from this pilot study will inform the sample size calculation and group allocation strategy for subsequent confirmatory studies.

The non-inclusion criteria of the study are: (i) practicing more than 3 h of swimming per week, (ii) use of anti-inflammatory drugs within 48 h prior to participation, and (iii) use of antibiotics and/or probiotics within 3 weeks prior to participation. The 3-hour/week swimming threshold was chosen to minimize the potential confounding effect of chlorinated pool water exposure on the oral microbiota, as regular swimming activity has been reported to alter the oral microbial composition (36).

Participants may also be excluded if they withdraw their consent or oppose the use of their data or biological samples (i.e., withdrawal of non-opposition). All eligible participants will receive both verbal and written information about the study. Written informed consent will be obtained before enrollment.

**Figure 1:**
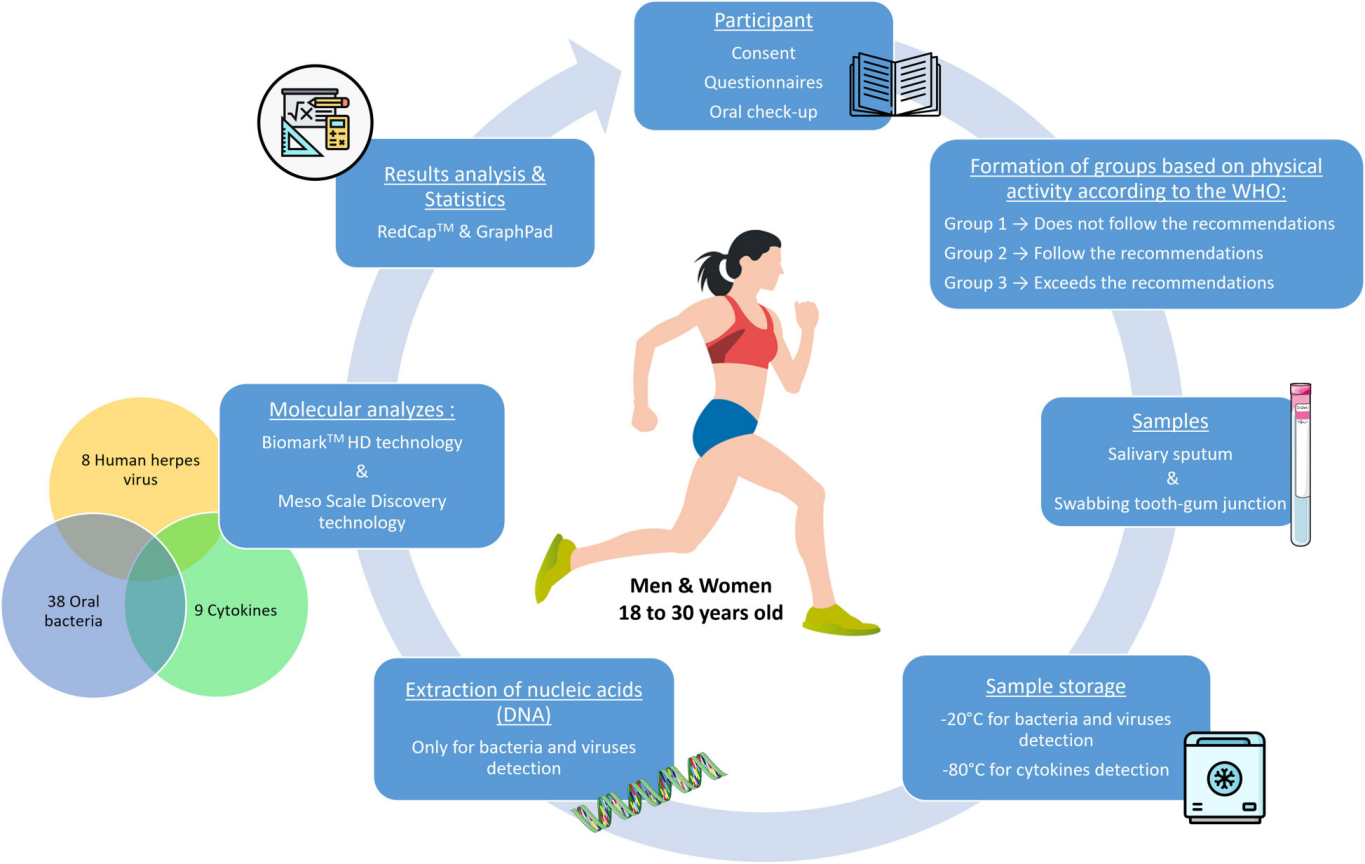
Schematic representation of the different stages of the ORASPORT pilot study. This figure outlines the complete recruitment process for participants in the ORASPORT study. Men and women aged 18 to 30 who express interest in participating will receive an information letter, a consent form, and a questionnaire to complete. The questionnaire includes items related to physical activity, diet, general health, and oral health. It is designed to collect essential baseline data and to classify individuals according to their physical activity level, based on World Health Organization (WHO) guidelines. All participants will undergo a standardized oral health assessment conducted by Dr. Margaux Dubois. During this clinical examination, two types of saliva samples will be collected: an unstimulated whole saliva and a swab from the mandibular region between gingiva and tooth. Depending on the sample type, they will be stored at either −20 °C or −80 °C in the laboratory until analysis. Genomic DNA will be extracted from the samples using Qiagen kits (see Materials and Methods) for the subsequent identification of bacterial and viral content using the Biomark HD™ system (Standard Biotools). Cytokines analysis will be performed directly on whole saliva samples without prior DNA extraction. Finally, statistical analyses will be conducted to explore correlations between oral bacterial profiles, human herpesvirus presence, cytokine levels, and participant characteristics according to their physical activity levels.

Figure 1 provides an overview of the study design and procedures.

### 2.3 Visit procedure

Each participant will attend a single study visit that includes the completion of a personal questionnaire, a comprehensive oral health examination, and the collection of oral biological samples.

#### 2.3.1 Personal questionnaire

Participants will complete a questionnaire designed to gather information across several domains linked to the study objectives. The questionnaire includes both validated tools and custom-designed sections and will be administered in paper format. It collects demographic data (age, sex, height, weight), medical and medication history, smoking status, and nutritional habits. In the nutritional habits section, the participants will be asked to report any specific dietary practices (e.g., vegetarian, vegan, gluten-free, lactose-free, flexitarian). To provide an overview of dietary behaviors, participants will be asked to self-identify the type(s) of diet they follow daily by selecting from a list of common dietary models (e.g., Mediterranean, Western, ketogenic, high-protein, high-carbohydrate, low-calorie). They will also be asked to estimate the relative frequency of adherence to each model, expressed as a percentage (totaling 100%). Additional items focus on sport-specific dietary habits, including the consumption of isotonic drinks (electrolyte-rich) drinks, energy drinks, energy gels, or energy bars. Participants will be asked to indicate the frequency, duration of use, and brand names where applicable.

Physical activity levels will be assessed using the International Physical Activity Questionnaire (IPAQ), which estimates overall activity in MET-minutes/week. Further details on sports-specific habits will also be recorded, including type, frequency, intensity, and duration of physical activities, as well as competition level and professional status. Participants will also report on their oral health status and behaviors, including the history of oral trauma, frequency of dental visits, and use of oral hygiene devices. Psychological and physiological stress levels will be assessed using Likert scales. Finally, the 14-item validated Oral Health Impact Profile (OHIP-14) will be employed to evaluate oral health-related quality of life.

The collected responses will help identify confounding factors and support subgroup classifications within the study cohort. All the data will be entered, centralized, and organized in a secure database for subsequent statistical analysis using the RedCap™ platform. Data entry will be performed by one of the two study investigators (MD or MO) on a password-protected computer in the MICORALIS laboratory, with access restricted and monitored. Examiners will be available during the questionnaire session to assist participants and answer any questions as needed.

#### 2.3.2 Oral health examination

Each participant's oral health will be assessed by the study's designated dentist (MD). Caries status will be diagnosed using both the Decayed, Missing, and Filled (DMF) index, following the WHO's 5th edition criteria, and the International Caries Detection and Assessment System (ICDAS) criteria (37, 38). Gingival status will be evaluated using the Löe and Silness Index (LSI) and the Plaque Index (PI) (39). Tooth wear and gingival recession will also be recorded, using Miller's classification system (40). Additional clinical observations will include the presence of orthodontic retainers, prosthetic restorations, tartar, and any other relevant dental conditions.

The clinician performing the oral examinations is blinded to the participants' group allocation. Group classification is conducted after the dental assessments, based on questionnaire data regarding physical activity.

#### 2.3.3 Oral sampling

Saliva samples will be collected immediately following the oral health examination. Participants will be instructed to refrain from eating, drinking (including water), toothbrushing, or smoking for at least 30 min before sampling. An unstimulated whole saliva sample (2 mL) will be obtained via passive drooling (spit method) into sterile tubes. Additionally, a mucosal smear will be collected by swabbing the junction between the tooth and gingiva in the mandibular region using a sterile swab.

All biological samples will be transported to the MICORALIS Laboratory for processing and storage. Each saliva sample will be divided into two 1 mL aliquots. The first aliquots, intended for microbiological analysis, will be mixed with a virus-inactivating and nucleic acid-stabilizing buffer and stored at room temperature until DNA extraction. Extracted DNA will then be stored at −20 °C prior to high-throughput quantitative PCR analysis using the Biomark HD™ system. The second aliquot, intended for immunological analysis, will be treated with protease inhibitors (Pierce™ Protease Inhibitor Tablets, Thermo Scientific™) and immediately stored at −80 °C until cytokine quantification is performed using Meso Scale Discovery® (MSD) multiplex immunoassay technology. Gingival swabs will be stored at 4 °C for a maximum of 24 h or directly frozen at −20 °C to preserve DNA integrity and minimize nuclease activity or microbial overgrowth.

### 2.4 Cytokines quantification

Cytokines levels will be measured using Meso Scale Discovery® MULTI-ARRAY® technology, an electrochemiluminescence-based patterned array system, offering high sensitivity, broad dynamic range, and multiplexing capability. The S-PLEX® kit was selected for its ability to simultaneously quantify 9 cytokines per sample with detection limits in the femtogram per milliliter (fg/mL) range. The procedure begins with washing the plate 3 times with 150 μL of PBS-T. Then, 50 μL of coating solution is dispensed into each well and incubated with shaking for one hour at room temperature. The plate is then washed, and 25 μL of blocking solution per well is added, followed by 25 μL of calibrator or sample. The plate is incubated at room temperature with shaking for 1.5 h. After this incubation, the plate is washed again, and 50 μL of TURBO-BOOSTTM antibody solution per well is added, followed by another 1 h incubation at room temperature with shaking. The second step, referred to as enhancement, involves adding 50 μL of enhancement solution to each well and incubating the plate at room temperature with shaking for 30 min. This is pursued by the addition of 50 μL of TURBO-TAGTM detection solution and a further 1-hour incubation at 27 °C with shaking. The final step, reading, consists of 3 additional washes with PBS-T and the addition of 150 μL of MSD GOLD Read Buffer to each well. The plate is then read immediately on an MSD instrument without further incubation.

### 2.5 Microbiological analysis

DNA will be extracted from unstimulated saliva samples using the QIAamp DNA Blood Midi kit® (Qiagen, Cat. No.: 51185) and from swab samples using the QIAamp DNA Mini Kit® (Qiagen, Cat. No.: 51304), following the manufacturer's protocols. DNA quantification will be carried out using a microvolume spectrophotometer (Nanodrop Lite Plus™), and concentrations will be normalized to 10 ng/μL for saliva samples and to 1 ng/μL for swab samples.

Detection and quantification of the various oral microbiological species (see Table 1 for details) will be performed using the Biomark HD™ technology. This high-throughput technology allows the simultaneous detection of dozens of microbial targets across a large number of samples (41). All experiments performed with the Biomark HD™ system will be conducted using 96.96 IFC plates, following the manufacturer's instructions and reagent kits (Standard Biotools. No.: BMKM10-96.96-EG) supplied by Standard Biotools. We adhered to the protocol “Preamplification of cDNA for Gene Expression with Delta Gene assays”. Preamplification was performed under the following cycling conditions: 95 °C for 2 min, followed by 12 cycles of 95 °C for 15 s and 60 °C for 2 min. After Exonuclease I digestion, samples will be diluted 1:20 and processed following the protocol “Gene expression with the 96.96 IFC using Delta Gene assays on preamplified samples”, using the AX SsoFastTM EvaGreen® Supermix with low ROX (Bio-Rad, 172-5211). Quantitative amplification was conducted on the Biomark HD™ system using a microfluidic real-time PCR protocol optimized for microbial detection. The reaction workflow began with a UNG (Uracil-N-Glycosylase) treatment at 50 °C for 2 min to eliminate potential carryover contamination, followed by a hot start activation phase at 95 °C for 10 min. PCR amplification comprised 40 cycles of denaturation at 95 °C for 15 s and annealing/extension at 60 °C for 60 s, with fluorescence data acquisition at each annealing step.

Each sample will be analyzed in duplicate, including both primer and sample replicates, using 1.25 μL of template DNA, corresponding to 12.5 ng of DNA for saliva samples and 1.25 ng for swab samples. The data were analyzed with Real-Time PCR Analysis Software using automatically defined thresholds. Negative samples were defined as those with Cq values ≥ 35 for each species. Final results will be normalized using standard curves and expressed as copy numbers per microgram (µg) of DNA.

### 2.6 Data visualization and analysis

The study will generate primarily quantitative data, including the abundance of each microorganism in saliva and mucosal samples, salivary inflammatory markers, oral health parameters (e.g., number of teeth, caries index, plaque index, gingival index, periodontal index), and variables derived from graded scoring system used to interpret data from health questionnaires. Data will be analyzed individually and compared across the three groups, classified according to their level of physical activity. The methodology is based on the development of an account matrix and the implementation of a relational database management system (RedCap™).

Descriptive statics will first summarize participant demographics and distributions. Univariate analysis will then be conducted using standard statistical tests appropriate for each variable (e.g., Student's t-test or Mann–Whitney *U*-test for continuous variables, ANOVA or Kruskal–Wallis for multiple-group comparisons, Chi-square tests for categorical variables, Pearson or Spearman correlation analysis).

To explore complex interactions between oral microbiota, immune markers, and clinical oral heath profiles, multivariate analysis such as principal component analysis (PCA) will be conducted. Subgroup comparisons based on qualitative criteria from self-reported questionnaires (e.g., dietary habits, smoking status) may also be considered. Potential confounding factors, including age, sex, smoking status, diet, and oral hygiene practices, will be accounted for using multivariate linear or logistic regression models, with covariates selected based on theoretical relevance and results from univariate analysis. This strategy ensures that observed associations reflect true relationships rather than confounded effects. Statistical significance will be set at *p* < 0.05.

## 3 Discussion

This study is based on the premise that oral health reflects overall systemic health. It specifically hypothesizes that regular physical activity, widely recognized for its profound effects on physiological functions, may lead to measurable changes in the balance of the oral ecosystem. While the impact of physical activity on both oral health and athletic performance are well documented (42–44), the influence of exercise on the oral microbiota remains poorly characterized (34). To our knowledge, this study is the first to systematically explore the effects of physical activity on oral health, oral microbiota, and immune response.

The lack of robust and validated biomarkers is particularly striking within the broader context of predictive genomics and personalized medicine. Biomarkers are measurable biological parameters associated with physiological or pathological processes (45). This study offers several notable strengths. It employs cutting-edge high-throughput molecular technologies to investigate both the oral microbiome and the immune system. A major technological innovation lies in the use of high-throughput microfluidic real-time PCR (Biomark HD™ system), which enables the simultaneous detection and quantification of up to 9,216 qPCR reactions in a single run. This platform allows parallel analysis of 96 biological samples across 48 microbiological targets in duplicate, combining high analytical efficiency with an optimized cost-to-performance ratio. Accordingly, a targeted panel of 48 microorganisms (bacteria and viruses) was selected based on recent literature and Socransky's bacterial complexes. This panel includes both commensal bacteria, indicative of oral health, and periodontopathogenic bacteria, associated with gingivitis and periodontitis. In addition to bacterial targets, 9 human herpesviruses were included in the panel, based on the growing evidence supporting the viral–bacterial synergy theory proposed by Slots (46). This concept suggests that herpesviruses may act as cofactors in the pathogenesis of periodontal diseases by modulating host immune responses and enhancing bacterial virulence. Recent studies have demonstrated potential associations between these viruses and the progression of periodontitis, further supporting their inclusion in the present analysis (47). Moreover, the characterization of salivary cytokines and immunoglobulins is performed using a multiplex electrochemiluminescence immunoassay (Meso Scale Discovery®, MSD), which enables the simultaneous quantification of up to 9 immune markers, in duplicate, across 40 samples per run. The S-PLEX® Proinflammatory Panel 1 (Human) was chosen for its high sensitivity in the femtogram range and a broad dynamic detection range. This panel includes cytokines representing both regulatory and pro-inflammatory pathways, allowing a balanced assessment of participants' overall immunological status.

This study also provides a unique opportunity to investigate the impact of physical activity on oral health in young adults with no underlying medical conditions. To our knowledge, it is the first to systematically examine how different levels of physical activity influence key oral health parameters, through the comparison of distinct groups within a large, well-defined cohort.

Several confounding factors, including dietary habits, oral hygiene practices, and psychological stress, are considered in the study to minimize bias in the observed associations. The questionnaire used represents a key strength, as it captures a wide range of lifestyle variables, including assessments of diet, sports-related nutritional supplementation, stress levels, and oral health behaviors. The questionnaire's structure, which combines self-administered validated items, reinforces the reliability and validity of the collected data. Overall, the methodological rigor and scientific relevance of the approach contribute to the robustness and interpretability of the findings. However, this pilot study also presents several limitations. As an exploratory investigation involving a relatively small sample size, its findings must be interpreted with caution. Due to the absence of prior studies on this specific research question, a robust sample size calculation was not feasible at the study's outset. Follow-up studies will therefore be required to establish reproducible, standardized assays and to validate candidate biomarkers in larger, prospective cohorts. Another limitation concerns the narrow age range of participants (18–30 years), which was intentionally selected to reduce age-related variability in oral microbiota composition. Furthermore, the study is limited to endurance sports. Other types of physical activity may exert distinct influences on the oral microbiota. For instance, the use of mouthguards in contact sports, such as boxing, or exposure to chlorinated water in aquatic disciplines, could introduce confounding variables affecting microbial profiles (48–50).

Ultimately, the identification of reliable biomarkers could significantly improve individualized health monitoring in both athletic and sedentary populations, enabling earlier detection of physiological disturbances and enabling the implementation of targeted, personalized health recommendations.

## 4 Patient and public involvement

The protocol for this pilot study was developed and coordinated by the principal investigators (MO, MD), with specific contributions from researchers at Université Côte d'Azur representing four complementary entities: MICORALIS, LAMHESS, IPMC, and the University Hospital of Nice (CHU). This transdisciplinary consortium brings together methodological expertise from odontology, microbiology, sports science, biology, and public health.

MICORALIS (UPR UCA 7354) specializes in the study of the etiopathogenesis of oral diseases and the relationships between oral microbiology and systemic health, supported by advanced technical platforms. LAMHESS (UPR6312 UCA) focuses on health-related and performance-oriented physical activity. IPMC (UMR UCA/CNRS 7275) is internationally recognized for its expertise in high-throughput molecular analysis of nucleic acids. The Odontology Department of the CHU de Nice, as the regional dental care provider, contributes to the oral health monitoring of study participants.

The study sponsor is the Clinical Research and Innovation Delegation of the Nice University Hospital. Any publication or communication must acknowledge this sponsorship and adhere to the institution's scientific authorship charter. Investigators are therefore required to submit any communications and/or manuscripts to the Research and Innovation Delegation for approval before dissemination.

## 5 Ethics and dissemination

### 5.1 Data collection and management

Free and informed non-opposition will be documented following article L-1122-1 of the French Public Health Code. Participants will be fully informed of their rights, including the right to refuse or withdraw from the study at any time, without justification. Before enrollment, a comprehensive explanation of the study protocol and its implications will be provided by the investigator or designated physician. Participants will also be informed of their entitlement to access aggregate results from the study. The non-opposition form is designed to ensure clarity and accessibility to individuals across varying levels of health literacy. A reflection period may be implemented between the dissemination of information and the collection of non-opposition. The investigator will present the non-opposition form in duplicate, retaining the original and providing a signed copy to the participant. Both the information leaflet and the non-opposition form are included in the study protocol appendices.

To ensure participant anonymity, all biological samples will be processed using a unique identifier, with tubes labeled with a numerical code corresponding to the participant's inclusion number and an alphabetical code designating the sample type.

### 5.2 Safety considerations

All personnel involved in the study are bound by medical and professional strict confidentiality. All documents and data related to the study will be stored in a secured, access-controlled environment.

### 5.3 Ethical considerations and dissemination

The study will be conducted in full compliance with French bioethics laws and all applicable legislative and regulatory provisions. This project is classified as a Category 3 human research study under the French Public Health Code (Law No. 2012-300 of March 5, 2012), as amended by Ordinance No. 2016-800 of June 16, 2016, and by the implementing decrees of the Jardé Law on Research Involving the Human Subjects (Decree No. 2016-1537 of November 16, 2016).

Ethical approval for this study was obtained from the Committee for the Protection of Persons (CPP) in February 2023. The approved protocol ensures both scientific rigor and adherence to ethical standards. Any substantial amendments to the study protocol will require prior approval from the CPP. Minor amendments will be communicated to all investigators and will require their agreement, as authorized by the principal investigator. The trial is registered in the national database managed by the French National Agency for the Safety of Medicines and Health Products (ANSM), under reference number RCB: 2022-A01682-41-22-PP-11.

This research complies with the amended French Data Protection Act (no. 78-17, January 6, 1978) and the European General Data Protection Regulation (GDPR, 2016/679). Data processing is registered with the Nice University Hospital and, in accordance with CNIL deliberation No. 2018-154 (MR-003), will follow the referenced methodology for health research that does not require explicit consent. Data collected before participant withdrawal will be retained and processed as described in the study protocol and in compliance with Articles 17.3.c and 17.3.d of the GDPR.

### 5.4 Status and timeline

Participant inclusion began on February 7, 2023, and is expected to be completed on April 15, 2026.
